# Minority Stress in the Context of the Disablement Process Model

**DOI:** 10.1093/geroni/igab046.3158

**Published:** 2021-12-17

**Authors:** Jeffrey Lentz

**Affiliations:** University of North Georgia, Oakwood, Georgia, United States

## Abstract

The changing demographics and growing diversity in the United States pose significant challenges for researchers, particularly scholarship involving sexual minority adults’ health and aging processes. Not much is known about how all minority stressors could lead to a disability. Sexual minority adults are at a greater risk of developing a disability later in life than their heterosexual counterparts (Fredriksen-Goldsen, Kim, & Barkan, 2012). Drawing from critical components of the disablement process model (Verbrugge & Jette, 1994), this dissertation sought to understand the relationship between minority stress and disability status among sexual minority adults 50 years and older. Minority stress in the context of the disablement process model is a social condition. While exploring the relationship between minority stress and disability status, intra-individual factors and extra-individual factors were assessed to see if they mediated the relationship between minority stress and disability status among sexual minorities 50 years and older. Discrimination is significantly associated with having a disability. None of the intra-individual factors and extra-individual factors mediated the relationship between minority stress and disability; however, several intra- and extra-individual were associated with greater or lesser odds of experiencing a disability. This dissertation concluded that discrimination is associated with disability status among sexual minority adults 50 years and older. On the other hand, the disablement process model does not support minority stress as a social condition leading to a disability. On the other hand, this dissertation's results support the ideology that experiencing discrimination is associated with a disability.

